# Effects of gratitude intervention on mental health and well‐being among workers: A systematic review

**DOI:** 10.1002/1348-9585.12290

**Published:** 2021-11-11

**Authors:** Yu Komase, Kazuhiro Watanabe, Daisuke Hori, Kyosuke Nozawa, Yui Hidaka, Mako Iida, Kotaro Imamura, Norito Kawakami

**Affiliations:** ^1^ Department of Mental Health Graduate School of Medicine The University of Tokyo Tokyo Japan; ^2^ Japan Society for the Promotion of Science Tokyo Japan; ^3^ Department of Public Health Kitasato University School of Medicine Sagamihara Japan; ^4^ Faculty of Medicine University of Tsukuba Tsukuba Japan; ^5^ Department of Psychiatric Nursing Graduate school of Medicine The University of Tokyo Tokyo Japan

**Keywords:** gratitude intervention, mental health, systematic review, well‐being, worker

## Abstract

**Objectives:**

Gratitude intervention, which requires participants to engage regularly in brief activities designed to cultivate a sense of gratefulness, is known as one of the most effective positive psychological interventions. Although numerous meta‐analyses and systematic reviews have been conducted on gratitude intervention, no studies have focused on the working population. This study aimed to systematically summarize the effectiveness of gratitude interventions on workers' mental health and well‐being.

**Methods:**

Systematic search was conducted in February 2021 using five databases. Eligible studies included randomized controlled trials implementing gratitude activities among healthy workers and measuring mental health or well‐being indicators and original articles or thesis in English.

**Results:**

Nine out of 1957 articles met the inclusion criteria. Eight studies adopted gratitude list interventions, showing a significant improvement in perceived stress and depression; however, the effects on well‐being were inconsistent. Interventions with gratitude list four times or less did not report significant changes in any outcomes.

**Conclusions:**

Most gratitude interventions incorporated a gratitude list, and some studies included gratitude activities as a part of the combined program. On the other hand, no studies focused on only behavioral gratitude expression among workers. Gratitude interventions might be effective in improving mental health, but their effects on well‐being remain unclear. The total number of gratitude lists and reflections might influence the effect on mental health and well‐being; however, due to the high heterogeneity of the studies, further studies are needed.

## 1 INTRODUCTION

Positive psychology has spent the past two decades investigating human strengths and virtues, solidifying the “science of positive subjective experience, positive individual traits, and positive institutions”.^1^ Since its inception, positive psychology has influenced various disciplines, including education, health care, and economics.^2^, ^3^ Positive psychology interventions have accumulated evidence of their effects on mental health and well‐being.^4^ In recent years, positive psychology interventions have also been introduced in the occupational health field. A meta‐analysis conducted in 2019 has demonstrated their effectiveness in improving work‐related outcomes, including job stress, engagement, and organizational prosocial behavior.^5^ Gratitude emerged from the study of positive psychology as a multidimensional concept involving an emotion, a personality trait, or a coping response.^6^ Especially in the academic context, much research has been done on the two concepts of gratitude: trait gratitude and state gratitude.^7^ Trait gratitude refers to the predisposition to be aware of situations in which one receives benefits from others and represents between‐person differences in the threshold to experience gratitude without specific events/experiences.^8^ Separately, gratitude as a state‐level emotion is a discrete experience that occurs when one perceives themselves as the recipient of a positive outcome, triggering a subsequent desire to reciprocate or otherwise engage in prosocial behavior.^9^, ^10^ Several studies have found an association between trait gratitude and more frequent and intense state gratitude experiences.^11^, ^12^ Many studies have demonstrated the positive moderate to large associations between gratitude and well‐being, such as positive affect, happiness, and life satisfaction.^7^, ^13^, ^14^ Regarding these mechanisms, Wood et al. introduced two gratitude specific hypotheses,^7^ (a) the schematic hypothesis (grateful people have characteristic schemas that influence their interpretation of situations more positively) and (b) the coping hypothesis (grateful people make more positive coping appraisals, and less likely to behaviorally disengage, deny the problem exists) along with two more general hypotheses, (c) the positive effect hypothesis (positive emotions, including gratitude, have a protective effect on various mental disorders, leading to improved well‐being) and (d) the broaden and build hypothesis (positive affective states broaden people's momentary thought‐action repertoires to help them develop additional resources to enhance long‐term well‐being).

Two strategies, called gratitude interventions, have been generally used to promote gratitude.^15^ One is a gratitude list (gratitude journal), which involves the participants making written lists of several things for which one is grateful regularly.^7^ Another strategy, a behavioral expression of gratitude,^7^ encourages the participants to express their grateful feelings to others.^7^ The most cited behavioral expression conducted by Seligman is called gratitude letter, in which participants write gratitude letters to their benefactors and read the letters to them.^16^ These interventions aim to increase state gratitude through activities; however, because state gratitude is a short‐term phenomenon that is difficult to assess, trait gratitude is often measured as an outcome. Gratitude interventions have several strengths. The objectives of the exercises are easy to understand and implement, as they are time and cost‐effective, tend to have lower dropout rates, and do not require experts in psychology.^15^, ^17^ The previous meta‐analyses and systematic reviews have indicated positive effects of gratitude intervention on well‐being (e.g., life satisfaction, happiness, and positive affect),^15^, ^17^ physical health (e.g., blood pressure, glycemic control, and inflammatory markers),^18^, ^19^ and mental health (e.g., depression and anxiety)^20^, ^21^ among various populations, including clinical, resident, and school.

Gratitude is also important for workers. A gratitude trait at work is defined as the tendency to recognize and be thankful for how various aspects of a job affect one's life.^22^ Worker's gratitude has a significant favorable correlation with well‐being (e.g., positive affect and life/job satisfaction), mental health (e.g., depressive symptoms and distress), and work‐related outcomes (e.g., job performance, organizational commitment, and citizenship behavior).^22^, ^23^ Furthermore, a meta‐analysis of positive psychology interventions among workers indicated that employee gratitude interventions for desirable work outcomes were shown to have stronger mean effect sizes (g = 0.34) compared to other interventions, such as psychological capital interventions or well‐being interventions, even though the differences between them were non‐significant.^5^ Although numerous meta‐analyses and systematic reviews have been conducted on gratitude intervention,^15^, ^17^, ^18^, ^19^, ^20^, ^21^ no studies have focused on the working population. Furthermore, since positive psychology and gratitude interventions at work have been suggested to be effective for mental health, well‐being, and work‐related outcomes,^5^ it would be worthwhile to qualitatively summarize the intervention methods and their effectiveness. In addition, gratitude intervention among workers may have characteristics varying from other populations. For example, workplace mindfulness training programs often differ from the standard protocols supported by scientific evidence.^24^, ^25^ These variations include reduced time commitment (or dose) of training and the use of flexible delivery methods to meet the demands of contemporary work environments.^26^ Similarly, gratitude intervention among workers might be tailored due to their limited time to devote to the working tasks. According to the review of 64 gratitude intervention studies,^18^ the most common intervention durations were 4 and 6 weeks, with some of them lasting from four to eight months. On the other hand, the gratitude intervention among workers conducted by Neumeier et al. lasted a week, arguing the importance of that it could be easily combined with various work schedules and could be flexibly integrated into daily work routines with relatively little effort.^27^ The findings relevant to the working population would be meaningful for developing and validating further gratitude intervention.

The current study aimed to systematically summarize randomized controlled trials to examine the effect of gratitude interventions on improving mental health and well‐being among workers. We searched the latest studies published until February 2021 to qualitatively summarize (1) the types of gratitude interventions conducted among workers, (2) the effectiveness of gratitude interventions in improving mental health and well‐being among workers, and (3) conditions and settings that are effective for improving mental health and well‐being among workers.

## 2 MATERIALS AND METHODS

### 2.1 Study design

The present study is a systematic review of randomized controlled trials (RCTs) to provide a qualitative summary of gratitude interventions implemented among workers and examine their effects on mental health and well‐being. This manuscript was written following the Preferred Reporting Items for Systematic Reviews and Meta‐Analyses (PRISMA) guidelines.^28^ The study protocol was registered at the UMIN Clinical Trials Registry (ID = UMIN000039785).

### 2.2 Eligibility criteria

Participants, interventions, comparisons, and outcomes (PICO) of the eligible studies were defined. Participants included all healthy workers. Interventions were defined as any interventions that included gratitude activities. Based on previous meta‐analyses,^15^, ^17^ we categorized gratitude interventions into three types, gratitude list, behavioral gratitude expression, and others (such as drawing a picture of something one is thankful for or taking psychoeducation). Three good things (TGT) exercise was also included as a gratitude list intervention, consistent with previous studies.^15^, ^17^ TGT exercise is similar to making the gratitude list, except that the participants are instructed to write down three good things that happened in a specified period.^16^ This is known as an activity to induce gratitude.^29^ Although the previous meta‐analyses excluded the mixed intervention that also contained other activities besides gratitude,^15^, ^17^, ^18^, ^19^, ^20^, ^21^ we broadly included these programs because we thought that including both would provide more practical knowledge. In this study, we defined mixed intervention as a program that contains gratitude and other activities and defined plain interventions as programs containing only gratitude activities. Comparison groups of the review were those conducted other activities or measurements only. Outcomes were mental health and well‐being indicators. In this study, mental health included anxiety, perceived stress, depression, and mental disabilities, such as burnout measured using standardized psychological symptom measures. Regarding well‐being, we included outcomes along with the definitions by Steptoe et al.^30^ Steptoe classified well‐being into three aspects, evaluative (how satisfied people are with their lives, such as job satisfaction and life satisfaction), hedonic (feeling or moods such as happiness or positive affect), and eudemonic (judgment about the meaning and purpose of life). In addition, eligible studies were (1) RCTs (adopting random assignment), (2) written in English, and (3) original articles or thesis.

### 2.3 Search and information sources

A systematic search was conducted in February 2021 using PubMed, Embase, PsycINFO, PsycARTICLES, and Web of science. The first author (YK) developed search terms based on the previous studies,^15^, ^17^, ^31^, ^32^, ^33^ and subsequently, coauthors discussed these terms and agreed with them. The search terms used were (1) keywords related to gratitude (e.g., gratitude, grateful, thankful, blessing), (2) participants (e.g., worker, employee, organization), and (3) study design (e.g., randomized controlled trial). The search terms are described in detail in Table 1.

**TABLE 1:** Search terms used in each database

Search database	Search term	Search day
Pubmed	(gratitude OR grateful* OR thankful* OR bless*) AND (employ* OR work* OR staff* OR personnel* OR supervis* OR team* OR manage* OR organizati* OR office* OR industr* OR compan* OR institut*) AND ((clinical[Title/Abstract] AND trial[Title/Abstract]) OR clinical trials[MeSH Terms] OR clinical trial[Publication Type] OR random*[Title/Abstract] OR random allocation[MeSH Terms] OR therapeutic use[MeSH Subheading])	2021/2/21
PsycINFO /PsycARTICLES	(gratitude OR grateful* OR thankful* OR bless*) AND (employ* OR work* OR staff* OR personnel* OR supervis* OR team* OR manage* OR organizati* OR office* OR industr* OR compan* OR institut*) AND (AB(clinical AND trial) OR clinical MJ trials OR PT (clinical trial) OR AB random* OR random MJ allocation OR MJ (therapeutic use))	2021/2/21
Web of science	(gratitude OR grateful* OR thankful* OR bless*) AND (employ* OR work* OR staff* OR personnel* OR supervis* OR team* OR manage* OR organizati* OR office* OR industr* OR compan* OR institut*) AND ((clinical AND trial) OR clinical trials OR random* OR random allocation OR therapeutic use)	2021/2/24
Embase	(gratitude OR grateful* OR thankful* OR bless*) AND (employ* OR work* OR staff* OR personnel* OR supervis* OR team* OR manage* OR organizati* OR office* OR industr* OR compan* OR institut*) AND ((clinical AND trial) OR clinical trials OR random* OR random allocation OR therapeutic use)	2021/2/22

### 2.4 Study selection

We entered all identified studies in a Microsoft^®^ Excel (Washington, USA) file. After YK excluded duplicate records, the remaining articles were distributed among the three authors (YH, MI, and KN), who independently in pairs assessed the title and abstract of each article to identify eligible studies according to the eligibility criteria (sifting phase). At this phase, we excluded studies that did not meet the eligibility criteria. In the next phase, a full‐text review was conducted. The pairs of investigators independently reviewed the full texts that passed the previous shifting phase. When the pairs of investigators disagreed then all investigators discussed and solved disagreements. The reasons for excluding studies were recorded during the full‐text review phase.

### 2.5 Data collection process and data items

Three investigators (YK, MI, and DH) independently extracted information from each included study. The year of publication, country, study design, the characteristics of the participants, response rate at baseline, follow‐up period after the intervention, follow‐up/dropout rate of the survey, the details of the gratitude intervention, the experimental and control conditions, outcomes, and the results of the mental health and well‐being were extracted. After extraction, all authors confirmed the collected information to reach a consensus in this process.

### 2.6 Risk of bias in individual studies

Three investigators (YK, MI, and DH) assessed the included study quality independently using the revised Cochrane risk of bias tool for randomized trials (RoB2),^34^ which evaluates randomized controlled study based on nine items: (1) random sequence generation, (2) allocation concealment, (3) blinding of participants and personnel, (4) blinding of providers, (5) blinding of outcome assessment, (6) blinding of data analysis, (7) incomplete outcome data, (8) selective reporting, and (9) other biases (e.g., cross over bias). Each item was then graded as high, some concerns, or low. Any discrepancies were settled by discussion among the investigators.

### 2.7 Synthesis of results

The data extracted from the included studies were summarized qualitatively. Based on the previous study,^17^ gratitude interventions were categorized into three types, gratitude list, behavioral expression of gratitude, and others. Gratitude intervention types were also categorized into two types, plain and mixed. The importance of considering the control conditions has been argued to rigorously discuss the effectiveness of the gratitude intervention.^7^ We categorized the control groups into three groups, positive, negative, and neutral, according to the previous study.^15^ Positive activities included performing random acts of kindness and identifying strengths, which were presumed to affect mental health and well‐being. Negative activities included listing daily/weekly hassles or misfortunes, which were presumed to affect the outcomes negatively. Neutral activities included listing daily/weekly activities, events, or measures‐only control, which were presumed to be psychologically inert. The consequences of the interventions were classified into three categories: significantly favorable effects (+), significant adverse effects (−), and insignificant effects (n.s.).

## 3 RESULTS

### 3.1 Study selection

**FIGURE 1:**
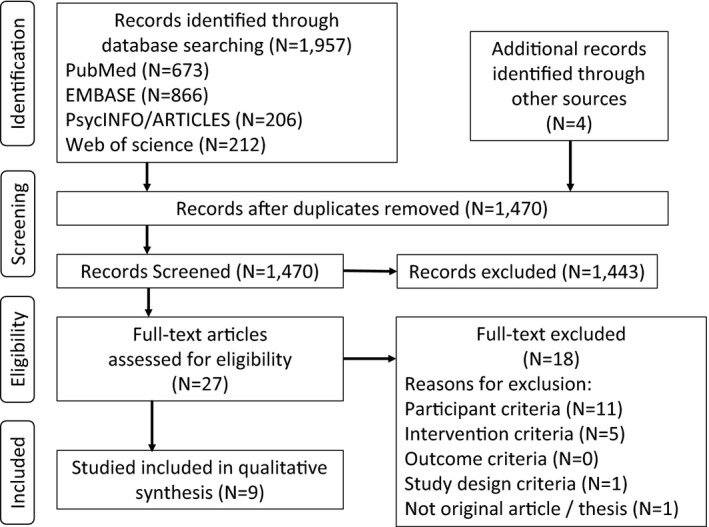
PRISMA flow diagram

Our initial search of five databases resulted in 1957 articles overall. After removing duplicates and adding four articles using a hand search, 1470 articles proceeded to the sifting phase. Among these, 1443 articles were excluded, and 27 articles proceeded to full‐text review. Following this process, nine articles were included in the qualitative review (Figure 1).

### 3.2 Study characteristics

The characteristics of the included studies are summarized in Table 2. Four gratitude intervention studies were conducted in the US^35^, ^36^, ^37^, ^38^ and three in China.^39^, ^40^, ^41^ One study was conducted in Japan^42^ and one in Australia.^27^ The participants included in the study were mostly health care professionals (*n* = 2)^39^, ^41^ or teachers (*n* = 2).^37^, ^40^ Four studies conducted a follow‐up survey after one week,^35^ one month,^36^, ^42^ and three months after the interventions,^41^ and the other five studies conducted a follow‐up survey immediately after the intervention.^27^, ^37^, ^38^, ^39^, ^40^ Regarding the response rate to the baseline survey (proportion of people who agreed to participate in the study out of the total number of people asked to participate), two studies had response rates above 80%,^39^, ^41^ and seven other studies did not report this. The follow‐up rates ranged from 50% to 97%. The completion rate of the intervention was not summarized in any studies.

**TABLE 2:** Design and settings of the studies included in the systematic reviews: *N* = 9

Study ID	Author, year	Country	Study design	Recruit	Dissemination for recruiting	Participants information (population, age, and sample size)	Response rate at baseline	Follow‐up period after intervention	Follow‐up /dropout rate
1	Ki, 2009.^39^	China, Hong Kong	RCT	A convenient sample.	NR	Health care professionals (nurse, doctor, physical therapist, and occupational therapist) Age: 18–50 (range) Sample size: 94 + 67 (men and women)	86% (180/210)	Immediate post‐intervention survey	89% (161/180)
2	Baker, 2011.^35^	The US	RCT	Conducted in a public university; only participants who had jobs and worked more than 10 h per week were recruited.	Participants enrolled in introductory psychology or a similar course were granted participation credit upon completion of the study.	Employees who, for the most part, were also undergraduate students in a public university. Age: 18–53 (range) 24 (mean for total) Sample size: 65 + 98 (men and women)	NR	1 week	92% (165/180)
3	Otsuka et al., 2012.^42^	Japan	RCT	Conducted at the mental health seminar held in local government.	NR	Daytime local‐government employees Age: 48.5 (mean for gratitude group) 48.4 (mean for the control group) Sample size: 28 + 9 (men and women), 1 missing	NR	1 month	50% (38/76)
4	Chan et al., 2013.^40^	China, Hong Kong	RCT	Invitation notice was posted on the author's teaching webpage to recruit volunteers.	To participate in an eight‐week self‐improvement project to enhance their well‐being through self‐reflection.	School teachers receiving in‐service training for postgraduate degrees Age: 22–58 (range) 33.7 (mean) Sample size: 15 + 63 (men and women)	NR	Immediate post‐intervention survey	96% (78/81)
5	Kaplan et al., 2014.^36^	The US	RCT	Various departments in two large public universities were recruited as potential participants.	The participants were told that the purpose of the study was to explore avenues to increase well‐being at work. Employees who completed the study could receive a $10 gift certificate for participating.	Staff members from two large public universities. Age: 43 (SD: 12.25) Sample size: 112 (sex was NR)	NR	1 month	60% (67/112)
6	Cheng et al., 2015.^41^	China, Hong Kong	Double‐blind RCT	The hospitals were chosen because of the availability of research assistants who were workers on‐site.	NR	Full‐time professional workers (physicians, nurses, physiotherapists, and occupational therapists) in 5 hospitals Age: NR Sample size: 46 + 56 (men and women)	82% (102/125)	3 months	97% (99/102)
7	Neumeier et al., 2017.^27^	Australia	RCT	Advertisements via social media, local newspaper, and radio directed participants to a website with information about the study and eligibility.	Aimed at testing two different online well‐being programs consisting of seven brief exercises to be completed during the next 7 days at work.	Employees Age: 19–73 (range), 41.7 (mean for PERMA group) 40.6 (mean for Gratitude group) 41.1 (mean for Wait list group) Sample size: 303 (sex was NR)	NR	Immediate post‐intervention survey	70% (303/431)
8	Cook et al., 2017.^37^	The US	RCT	A flyer was distributed by central administrative staff to secondary teachers within the school district.	The flyer offered access to free web‐based training for staff interested in learning skills to manage job‐related stress effectively and enhancing their overall well‐being.	High & middle school teachers from a single educational service district Age: NR Sample size: 44 (sex was NR)	NR	Immediate post‐intervention survey	81% (44/54)
9	Ligon, 2019.^38^	The US	RCT	The study sample was recruited from one mental health call center located in Iowa and three customer service call centers in Ohio, Florida, and California.	The purpose of the research was to examine whether three different work activities effectively reduce stress and increase hope, positive thinking, mental toughness, and confidence.	Employees of mental health and customer service call centers worked either a day shift or night shift full‐time (i.e., 40 h per week). Age: 18–29: 49 (sum of 3 groups) 30–39: 51 (sum of 3 groups) 40–49: 34 (sum of 3 groups) 50 or older: 14 (sum of 3 groups) Sample size: 55 + 89 + 4 (men and women and transgender)	NR	Immediate post‐intervention survey	94.9% (148/156)
	

Abbreviations: NR, Not reported; RCT, Randomized controlled trial.

### 3.3 Intervention strategies

Table 3 shows the summary of intervention methods and their effect on outcomes. Nine interventions reported in eight studies adopted gratitude list,^27^, ^35^, ^36^, ^38^, ^39^, ^40^, ^41^, ^42^ while one study conducted psychoeducational group sessions.^37^ No studies conducted incorporated only behavioral gratitude expression among workers. In studies using gratitude lists, six of eight studies asked participants to record "work‐related gratitude".^27^, ^35^, ^36^, ^39^, ^41^, ^42^ Five studies were web‐based,^27^, ^35^, ^36^, ^39^, ^40^ two studies were paper‐based,^41^, ^42^ and in the remaining studies, the participants could choose one of the two.^38^ Ki incorporated a web‐based gratitude list to 161 health care workers, including nurses, doctors, physical therapists, and occupational therapists, in China.^39^ Participants were asked to write down gratitude lists twice a week for 4 weeks, totaling eight lists. A negative activity was offered to the control group, which asked the participants to write down at least one hassle event at work.

**TABLE 3:** Interventions, outcomes, and results of the studies included in the systematic reviews: *N* = 9

Study ID	Author/ Year	Type	Duration & Frequency/Program hours	Gratitude conditions (plain/mixed)	Control conditions (negative/neutral/positive)	Outcomes	Results on mental health (+, −, n.s.)	Results on well‐being (+, −, n.s.)
1	Ki, 2009.^39^	Gratitude list (web‐based)	Twice a week for 4 weeks in both conditions (8 times).	Writing down at least one grateful event at work. (plain)	Writing down at least one hassle event at work. (negative)	Perceived stress: PSS PA and NA: CAS Life satisfaction: SWLS Depression: CES‐D 10	Perceived stress: + Depression: +	Positive affect: + Negative affect: + Life satisfaction: +
2	Baker, 2011.^35^	Gratitude list (web‐based)	Once a week, for 4 weeks (4 times).	Reporting four positive events that occurred during the week while at work or related to their job. (plain)	Measurement only(neutral)	Life satisfaction: SWLS PA and NA: PANAS Job satisfaction: JIG	NA	Life satisfaction: n.s. Positive affect: n.s. Negative affect: n.s. Job satisfaction: n.s.
3	Otsuka et al., 2012.^42^	Gratitude list (paper‐based)	Once a week, for 4 weeks among both conditions (4 times).	Writing down up to five people at work or in one's personal life to whom the participant was grateful during the past week in a journal. (plain)	Writing down up to five events occurred at work or in one's personal life during the past week in a journal for 4 weeks. (neutral)	PA: PANAS Life satisfaction: SWLS Subjective happiness: SHS	NA	Positive affect: n.s. Life satisfaction: n.s. Happiness: n.s.
4	Chan et al., 2013.^40^	Gratitude list (web‐based)	Once a week for eight weeks among both conditions (8 times).	Writing down up to three good things or events that happened to the participants. Setting at least 15 min at the end of the week to think about the meanings of these events. (plain)	Writing down up to three bad things or events that happened to the participants. Setting at least 15 min at the end of the week to think about the meanings of these events. (negative)	Life satisfaction: SWLS PA and NA: PANAS	NA	Life satisfaction: n.s. Positive affect: n.s. Negative affect: +
5	Kaplan et al., 2014.^36^	Gratitude list (web‐based)	Three times a week for 2 weeks (6 times).	Logging in at least three times per week to record things they are grateful for related to their job. (plain)	Engaging in specific strategies to increase their social ties at work social and document those experiences on a secure Web site. (positive)	PA and NA: JAWS	NA	Positive affect: + Negative affect: n.s.
6	Cheng et al., 2015.^41^	Gratitude list (paper‐based)	Twice a week for 4 weeks among both conditions (8 times).	Writing diaries about work‐related thankful events. (plain)	There are two control conditions. Control A: Writing diaries about work‐related hassle events. (negative) Control B: Measurement only (neutral)	Depression: CES‐D 10 Perceived stress: PSS	Compared with Control A, Depression + Perceived stress: + Compared with Control B: Depression: + Perceived stress: +	NA
7A	Neumeier et al., 2017.^27^	Gratitude list (web‐based)	During the following 7 days at work (7 times).	Writing down three things about work or workplace for which the participants genuinely feel grateful and reflect on them. (plain)	Measurement only (neutral)	Happiness: Eight well‐being indicators^a^ Happiness at work: eight well‐being indicators at work^b^	NA	Happiness: + Happiness at work: +
7B	Neumeier et al., 2017.^27^	Gratitude list (web‐based)	During the following 7 days at work (7 times).	Conducting a positive intervention program containing seven exercises (practicing gratitude, savoring the moment, you at your best, random acts of kindness, visualizing your best possible self, wearing a smile, and brainstorming meaningfulness). (mixed)	Measurement only (neutral)	Happiness: Eight well‐being indicators^a^ Happiness at work: eight well‐being indicators at work^b^	NA	Happiness: + Happiness at work: +
8	Cook et al., 2017.^37^	Others	2.5 h × 5 times in both conditions	Compounded program based on positive psychology consisting of eight practices: practicing gratitude (writing and delivering a gratitude letter, gratitude journaling, and making thank you notes), mindfulness‐based practice, helping and doing good deeds for others. (mixed)	Participate in the controlled program to discuss topics related to their daily work. (neutral)	Perceived stress: PSS Job satisfaction: SWWS	Perceived stress: +	Job satisfaction: +
9	Ligon, 2019.^38^	Gratitude list (web‐based and paper‐based)	Each group completed the assigned task for 10 min once per week on the day of their choice for 2 weeks at work (twice).	Participants in the gratitude group were asked to recount and then write about three lifetime events for which they were grateful. (plain)	Control A: The optimism intervention involved writing about one's best possible future self (positive). Control B: The control condition involved writing about typical daily work activities (neutral).	Burnout: MBI‐GS	Burnout: n.s. (Result of comparing Gratitude condition and Control B)	NA

Note:

+, significant favorable effects; −, significant adverse effects; n.s., non‐significant effect; NA, not applicable.

The difference between gratitude interventions between study ID 5A and 5B in general/Work‐related subjective well‐being was non‐significant.

Summary of the scales and other information. CAS, Chinese Affect Scale^65^; CES‐D 10, Center for Epidemiologic Studies Depression Scale^67^, ^68^; JAWS, Job‐Related Affective Well‐being Scale^72^; JIG, Job In General scale^70^; MBI‐GS, Maslach Burnout Inventory^74^; NA, Negative affect; PA, Positive affect; PANAS, Positive and negative affect schedule^69^; PSS, Perceived Stress Scale^64^; SHS, Subjective Happiness Scale^71^; SWLS, Satisfaction with Life Scale^66^; SWWS, Satisfaction with Work Scale.^73^

^a^ Calculated by happiness, satisfaction with life and positive and negative affect.^27^

^b^ Calculated by happiness at work, job satisfaction and positive and negative affect at work.^27^

Baker incorporated a web‐based gratitude list to 163 employees recruited from a public university in the US.^35^ Participants were asked to wire four gratitude lists related to their job once a week for 4 weeks. The study adopted a neutral control group that measured the outcomes only.

Otsuka et al. incorporated a paper‐based gratitude list to 38 employees in a local government in Japan.^42^ Participants were asked to write four gratitude lists once a week for 4 weeks. They listed five people at work or in one's personal life to whom the participant was grateful during the past week. The study set a neutral comparison that asked the participants to write up to five events at work or in one's personal life during the past week in a journal for 4 weeks.

Chan et al. incorporated a web‐based gratitude lists to 78 schoolteachers receiving in‐service training for postgraduate degrees in China.^40^ The author disseminated that this is an eight‐week self‐improvement project to enhance their well‐being through self‐reflection. Participants were asked to write three good things or events that happened to the participants once a week for 8 weeks. They also had at least 15 min to think about the meanings of these events at the end of the week. The control group was offered a negative activity that asked the participants to write down three bad things or events that happened to the participants and think about these events' meanings.

Kaplan et al. incorporated a web‐based gratitude list to 112 staff members from two large public universities in the US.^36^ The participants were told that the purpose of the study was to explore avenues to increase well‐being at work. Participants were asked to create six gratitude lists, recording grateful things related to their job three times a week for 2 weeks. The control group completed a positive activity that asked the participants to engage in specific strategies to increase their social ties at work three times per week and document those experiences on a secure Web site.

Cheng et al. incorporated a paper‐based gratitude list to 102 health care workers, including physicians, nurses, physiotherapists, and occupational therapists recruited in five hospitals in China.^41^ Participants were asked to create eight lists by writing diaries about work‐related thankful events twice a week for 4 weeks. Two control groups were included. One group completed a negative activity, writing diaries about work‐related hassle events (Control A), while the neutral comparison group completed only the measurements (Control B).

Neumeier et al. incorporated a web‐based gratitude list to 303 participants recruited via social media, newspapers, and radio in Australia^27^ and assigned to three groups (plan gratitude group, mixed gratitude group, and neutral control group). In the plan gratitude group, participants were asked to create seven lists by writing down grateful things about work or the workplace. Subsequently, they reflected on the grateful events during the following 7 days at work. In the mixed gratitude group, participants were asked to complete seven positive exercises during the consecutive 7 days at work: "practicing gratitude” (the same exercise the plain gratitude group), "savoring the moment” (mindfully savoring a pleasurable activity by paying attention to your immediate experience), “you at your best” (writing a story about a time when you were at your best at work and reflecting on your personal strengths displayed in the story), “random acts of kindness” (performing three acts of kindness in your workplace to benefit others or make others happy), “visualizing your best possible self” (thinking and writing about your best possible professional self/working life and defining specific goal that would help you to attain this best possible future scenario), “wearing a smile” (relaxing, finding something that makes you laugh, and frequently wearing a smile over the day), and “brainstorming meaningfulness” (brainstorming about tasks or elements in your work that you find meaningful or that are significant to you, and creating a mind map about sources of meaningful experiences in your job). The participants in the control group completed only the measurements.

Cook et al. conducted psychoeducation that included gratitude activities in group sessions.^37^ The participants were 44 high and middle school teachers from a single educational service district in the US. They completed five 2.5‐h group sessions. The program was developed as an intervention promoting the well‐being of teachers, helping them become resilient educators by focusing on eight practice areas: (1) increasing awareness and empowerment through mindfulness‐based practices, (2) paying attention to the positive and practicing gratitude, (3) helping and doing good deeds for others, (4) identifying unhelpful thoughts and altering them to be more helpful, (5) developing good sleep habits, exercising regularly, and eating well, (6) clarifying values and committing to them, (7) establishing good social support, role models, and a mentor (relationships), and (8) rewarding oneself through relaxation and recreation. Practicing gratitude requires three specific activities: (1) writing and delivering a gratitude letter, (2) weekly gratitude journaling that identifies 3–5 things one is grateful for and why, in addition to imagining what the week would be like if the things did not happen, and (3) paying attention to the small things and writing thank you notes to life (e.g., thankful for being able to take a warm shower and getting clean because it makes me feel better; thankful for being able to take walks and having time to think and getting healthy all at once). The control group underwent five neutral 2.5‐h group sessions to discuss topics related to their daily work.

In a more recent study, Ligon incorporated gratitude list to 148 mental health and customer service call centers in the US.^38^ It was both allowed to make gratitude list by paper‐based or web‐based. The participants were told that the purpose of the study was to examine whether three different work activities effectively reduce stress and increase hope, positive thinking, mental toughness, and confidence. The study lasted for 2 weeks, and the participants were asked to create two gratitude lists, one for each week, spending 10 min each week on the day of their choice to write about three lifetime events for which they were grateful. The comparison groups completed two other activities. In the positive activity group, the participants wrote about one's best possible future self once a week for 2 weeks. In the neutral group, they wrote about typical daily work activities once a week for 2 weeks.

### 3.4 Effects of the intervention programs on the outcomes

The included study adopted various mental health and well‐being outcomes. Mental health included perceived stress (*n* = 3),^37^, ^39^, ^41^ depression (*n* = 2)^39^, ^41^ and burnout (*n* = 1).^38^ Well‐being encompassed positive affect (*n* = 5),^35^, ^36^, ^39^, ^40^, ^42^ negative affect (*n* = 4),^35^, ^36^, ^39^, ^40^ life satisfaction (*n* = 4),^35^, ^39^, ^40^, ^42^ job satisfaction (*n* = 2),^35^, ^37^ happiness (*n* = 2),^27^, ^42^ and happiness at work (*n* = 1).^27^ Perceived stress and depression improved significantly in all three studies,^37^, ^39^, ^41^ while burnout did not.^38^ Furthermore, positive affect increased significantly in two studies^36^, ^39^ but did not change in three studies.^35^, ^40^, ^42^ Negative affect declined significantly in two studies^39^, ^40^ but did not change in two studies.^35^, ^36^ Life satisfaction increased significantly in one study^39^ but not in three studies.^35^, ^40^, ^42^ Job satisfaction significantly increased in one study^37^ but did not change in another study.^35^ Happiness and happiness at work increased significantly in Neumeier et al. ^27^ but not in Otsuka et al.^42^ No significant adverse effects were observed.

### 3.5 Effects of the gratitude list by duration and frequency of the programs

In the studies that adopted gratitude list as the intervention,^27^, ^35^, ^36^, ^38^, ^39^, ^40^, ^41^, ^42^ the number of gratitude lists ranged from two to eight. The frequency with which the participants wrote gratitude lists ranged from once a week to every seven workdays. The duration of the interventions ranged from 7 days to 8 weeks. The most commonly adopted intervention duration was 4 weeks.^39^, ^41^, ^42^ The studies that did not report significant changes on any outcomes required the participants to complete gratitude lists four times or less during intervention.^35^, ^38^, ^42^ The other studies reported significant improvement on at least one of the outcomes.^27^, ^37^, ^39^, ^40^, ^41^

### 3.6 Effects of the intervention by types of control groups (positive, negative, and neutral)

One of the included studies offered positive activities to the control group to increase their social ties at work and yielded inconsistent results on well‐being (significant for positive affect and insignificant for negative affect).^36^ Neumeier et al. compared plain gratitude intervention and mixed gratitude intervention with no significant differences in well‐being (happiness and happiness at work).^27^ In the study conducted by Ligon, although the positive intervention was provided, the intervention effect was not compared with the gratitude intervention group.^38^ Three of the included studies offered negative activities that asked participants to regularly write down hassle or bad events.^39^, ^40^, ^41^ The setting of these control groups was consistent with the most cited gratitude intervention study implemented by Emmons et al.^6^ The studies showed significant improvement in mental health (perceived stress and depression) and inconsistent results for well‐being. Specifically, Ki showed a significant increase in positive affect, negative affect, and life satisfaction^39^ Chan et al. showed no effects on positive affect and life satisfaction and reported a significant decrease in negative affect,^40^ while Cheng et al. showed a significant decrease in depression and perceived stress.^41^ However, the effectiveness may have been exaggerated because gratitude's benefits, adverse effects of negativity, or both might have maximized the observed differences.^15^

Six studies adopted neutral control.^27^, ^35^, ^37^, ^38^, ^41^, ^42^ In one neutral condition, the participants were asked to write down events that occurred at work or in one's personal life regularly or to participate in group sessions to discuss topics related to their daily work, while the other neutral condition included only the measurement. These two studies showed significant improvement in perceived stress and depression^37^, ^41^ and non‐significant improvement in burnout^38^ while the results on well‐being were inconsistent. Two studies reported insignificant changes,^35^, ^42^ and two studies reported a significant increase in job satisfaction,^37^ happiness, and happiness at work.^27^

### 3.7 Risk of bias within studies

Table 4 summarizes the risk of bias assessed using the revised Cochrane risk of bias tool for randomized trials (RoB2).^34^ Cheng et al.^41^ rated random sequence generation, allocation concealment, and incomplete outcome data as low risk and selective reporting as some concerns. In the study by Ligon,^38^ selective reporting was rated as low risk, while random sequence generation was rated as some concerns. All other domains of the studies were rated as high risk, resulting in all nine studies being rated as high risk of bias overall.

**TABLE 4:** Risk of bias, assessed by the revised Cochrane risk of bias tool for randomized trials (RoB 2): *N* = 9

Study	Random sequence generation	Allocation concealment	Blinding of participants and personnel	Blinding of providers	Blinding of outcome assessment	Blinding of data analysis	Incomplete outcome data	Selective reporting	Other bias	Overall
Ki, 2009.^39^	High	High	High	High	High	High	High	High	High	High
Baker, 2011.^35^	High	High	High	High	High	High	High	High	High	High
Otsuka et al., 2012.^42^	High	High	High	High	High	High	High	High	High	High
Chan et al., 2013.^40^	High	High	High	High	High	High	High	High	High	High
Kaplan et al., 2014.^36^	High	High	High	High	High	High	High	High	High	High
Cheng et al., 2015.^42^	Low	Low	High	High	High	High	Low	Some concerns	High	High
Neumeier et al., 2017.^27^	High	High	High	High	High	High	High	High	High	High
Cook et al., 2017.^37^	High	High	High	High	High	High	High	High	High	High
Ligon, 2019.^38^	Some concerns	High	High	High	High	High	High	Some concerns	High	High

## 4 DISCUSSION

This study systematically reviewed gratitude intervention studies on mental health and well‐being among workers. Many studies were conducted with health care professionals and teachers, while only a few studies were conducted with general workers. Most gratitude interventions were incorporated a gratitude list. No studies focused on only behavioral gratitude expression among workers. Although the studies consistently showed significant improvement in perceived stress and depression, effects on well‐being were inconsistent. The studies that did not report any significant changes in the outcomes instructed the participants to create four gratitude lists or less during the intervention. The other studies reported significant improvement in at least one of the outcomes. The most frequently adopted intervention duration was 4 weeks, consistent with the previous meta‐analysis.^18^ Three out of nine studies included negative activity groups, such as recording bad events, as a comparison. Compared to other populations, no distinct differences in the frequency or duration of the interventions were observed, although the characteristics of the recorded objects differed, with most studies asking the participants to record "work‐related" gratitude. This study updated the evidence of gratitude interventions by adding three studies^27^, ^37^, ^38^ that were never included in the previous review studies.

### 4.1 What kind of gratitude interventions were conducted among workers?

Consistent with the previous meta‐analysis,^17^ gratitude list was the most common strategy adopted with workers, seen in eight of nine studies. This is known as the classic and basic gratitude intervention.^6^ This approach may be suitable even for busy workers because it is easy to understand and complete, without much time or special materials.^15^ It was observed in both cases that less frequently completed gratitude list over a longer period (once a week for 8 weeks)^40^ and more frequently completed gratitude list over a shorter period (daily for a week).^27^ The follow‐up rate for each was over 70%, indicating that both methods are acceptable for workers.

On the other hand, behavioral gratitude expression was only included as part of a combined positive psychological program,^37^ there were no studies that incorporated behavioral gratitude expression only. This may be attributed to the difficulty of implementing it. The previous study found that college students felt less adept at writing a gratitude letter compared to keeping a gratitude list, which in turn predicted lower rates of completing the activities.^43^ Similarly, workers may hesitate to participate in gratitude intervention, including expressing their grateful feelings to others. However, a previous study utilizing the RCT design reported that outcomes were significantly improved in the group that combined gratitude list and behavioral gratitude expression compared to the group that completed only the gratitude list.^44^ To introduce behavioral gratitude expressions to workers, it may be necessary to provide a practice guide for conducting the activity. Cook et al. provided instructions to include a specific person to give the gratitude letter to, concrete steps for writing, the approximate number of words (~300 words), and steps for delivering the letter.^37^ Among workers, gratitude activities were often incorporated on a stand‐alone basis, but a few studies included them as part of a combined intervention, showing a high degree of adaptability in implementation methods.

### 4.2 Do gratitude interventions effectively improve mental health and well‐being among workers?

Gratitude interventions for workers might effectively improve perceived stress and depression; however, the effects on well‐being might be unclear. The effects of depressive symptoms on mental health outcomes were consistent with a meta‐analysis conducted by Cregg et al. among the general population.^21^ Three possible mechanisms have been discussed. First, gratitude was associated with interpreting various stimuli and life events in positive terms, which is inconsistent with the selective attention to negative qualities of the self, the world, and the future that characterize depression and anxiety.^45^, ^46^ Second, it was argued that a less critical, less punishing, and more compassionate view of oneself account for the inverse relationship between gratitude and symptoms of depression and anxiety.^47^ Finally, researchers have also found an association of gratitude with greater relationship connection and satisfaction,^48^ well‐established buffers against psychopathology.^49^ The basis of these mechanisms (interpretation of events, views of oneself, relationship connections, and satisfaction) is also closely related to workers, leading to consistent results. On the other hand, the effect of the gratitude intervention on burnout was not consistent with a previous pre‐post single‐arm study among workers (teachers).^50^ It was argued that the symptoms of burnout would be reduced when they experience professional growth, self‐efficacy, and perceived success in their career progression.^51^ More research is needed to conclude the effect of gratitude intervention on burnout.

Regarding well‐being, contrary to previous meta‐analyses,^15^, ^17^ inconsistent results were obtained. The most critical reason would be the high heterogeneity of intervention methods. The intervention effects on well‐being tended to depend on the intervention method rather than on the kind of well‐being indicators. In other words, effective studies showed improvements in multiple measured outcomes, while less effective studies failed to show any significant effects. To consider the effects of gratitude intervention on well‐being among workers, it is necessary to pay attention to whether effective conditions and settings are adopted, in addition to taking care of the control group (assigned activities are positive or negative, or neutral), as pointed out in the previous study.^7^The existing studies introduced various theories to explain the mechanism underlying the relationship between gratitude intervention and mental health or well‐being outcomes. For example, Ki, Baker et al, and Neumeier et al. cited the Broaden and Build Theory.^27^, ^35^, ^39^, ^52^ Additionally, Cheng et al. proposed the coping hypothesis,^41^ while Kaplan et al. explained applied the model of happiness (happiness is a function of three major factors: life circumstances, temperament/disposition, and positive cognitive or behavioral activities).^36^, ^53^ Since the gratitude intervention can affect multiple dimensions, including cognition, mood, behavioral tendencies, coping, traits such as prosociality, and relationships with others,^7^, ^17^, ^32^ several mechanisms may have a combined effect among workers as well.

### 4.3 What conditions and settings effectively improve mental health and well‐being among workers?

A key moderator of positive psychology interventions is the number of times a participant engages in an activity.^17^, ^54^ For example, in prior work in positive psychology, more time spent working on forgiveness activities resulted in larger effect sizes for forgiveness.^55^ From this perspective, the total number of gratitude lists might affect outcomes among workers differently. Studies that recorded gratitude lists six times or more showed significant effects at least on one outcome,^27^, ^37^, ^39^, ^40^, ^41^ while studies that recorded gratitude four times or fewer did not show significant results on any outcomes.^35^, ^38^, ^42^ This can be explained by the schematic hypothesis of gratitude introduced by Wood et al. as the mechanism underlying the relationship between gratitude and well‐being.^7^ The hypothesis suggests that grateful people go around in life with a particular interpretive lens, seeing help as more costly, valuable, and altruistic. Equally, ungrateful people view the help they see as lower on these dimensions. According to the Network Theory of Emotion,^56^, ^57^ emotional schemas develop linearly through repeated pairings of stimuli and emotions.^58^ The schema hypothesis of gratitude has also been supported in an occupational context.^59^ Thus, for a grateful schema, "repeated stimulation" would be necessary. Accordingly, less than four activities would not suffice. Whether the intervention duration is one week^27^ or 8 weeks^40^ or whether the frequency is high (daily) or low (once a week) does not seem to affect the formation of schemas, as long as the total number of gratitude activities are sufficient.

Considering differences in intervention content, six out of eight gratitude list studies asked participants to record "work‐related gratitude" while the remaining studies did not. In this study, the effect of these differences on mental health and well‐being has been inconsistent. However, whether the gratitude is work‐related might affect its effects. The gratitude list can also function as a reframing, specifically, positive reappraisal.^60^ Therefore, it may be desirable to promote positive reappraisal in the work domain, especially when targeting work‐related outcomes. In a study conducted by Ligon, where gratitude lists were not "work‐related" but "life‐related," no significant improvement in burnout was found.^38^ In the future, it will be important to investigate the relationship between the content of the gratitude recorded and the outcome. As another point in the gratitude list studies, both paper‐based and web‐based interventions were present, but it was seemed to be inconsistent differences in effectiveness by intervention medium. This is in line with the previous study that found no significant difference in performance between paper‐based and web‐based homework assignments among students.^61^ It would be desirable to choose a medium depending on employees’ work style to reduce the burden on workers.^35^ Two studies required the participants to reflect on and keep gratitude lists, and both showed improvement in one or more of the outcomes.^27^, ^40^ Reflecting on the gratitude list may enhance the intervention effect by "savoring." Savoring is a construct in positive psychology that refers to using one's cognitive or behavioral responses to regulate the emotional effect of positive events.^62^ The previous diary study showed that savoring mediated and moderated the effect of daily positive events on happiness and mood.^63^

Therefore, it might be useful to incorporate elements of savoring into gratitude intervention. In conclusion, the total number of gratitude lists and reflections might influence the effect on mental health and well‐being; however, due to the high heterogeneity of the studies (the content of the intervention, timing of measurement, subjects, etc.), further studies are needed.

### 4.4 Risk of bias within studies applying gratitude interventions

Based on our assessment of the risk of bias, all nine studies were rated as high risk of bias overall. While bias can occur in some domains due to the nature of the intervention (e.g., blinding of participants, personnel, and provider), it is necessary to study higher quality RCTs targeting workers. For example, randomization should be done by independent researchers, the process should be clearly stated, intention to treat (ITT) analysis should be employed, and protocol papers or registries should be opened in advance.

### 4.5 Limitations

The present systematic review has several limitations. First, we did not conduct a meta‐analysis because it was deemed inappropriate due to the large variability in mental health and well‐being indicators. Therefore, it is not possible to quantitatively verify the effects of the gratitude intervention among workers. Second, this review was limited to studies written in the English language. Third, it is possible that there was overlooked mixed intervention regardless they include gratitude activities substantially due to the reason it was not mentioned as gratitude activities clearly in the paper. Fourth, additional unpublished studies, especially those with negative consequences, were omitted. Therefore, publishing bias could not be ruled out. Fifth, generalizations have been limited because many studies were conducted on health care professionals and teachers, and few studies were conducted on general workers.

## 5 CONCLUSIONS

Most gratitude interventions incorporated a gratitude list, and some studies included gratitude activities as a part of the combined program. On the other hand, no studies focused on only behavioral gratitude expression among workers. Although studies in this review showed significant improvement in perceived stress and depression, the effects on well‐being were inconsistent. The total number of gratitude lists and reflections might influence the effect on mental health and well‐being; however, due to the high heterogeneity of the studies, further studies are needed.

## DISCLOSURE

*Ethical approval*: Ethical approval is not needed because data from previous studies in which informed consent was obtained by primary investigators were retrieved and included. *Informed consent*: N/A. *Registry and the Registration No. of the study/Trial*: The study protocol was registered at the UMIN Clinical Trials Registry (ID = UMIN000039785). *Animal Studies*: N/A. *Conflict of interest*: Yu Komase, Kazuhiro Watanabe, Daisuke Hori, Kyosuke Nozawa, Yui Hidaka, Mako Iida, Kotaro Imamura declare no competing interests. Norito Kawakami reports grants from Fujitsu LTD., SB At Work Corp., personal fees from Occupational Health Foundation, Japan Dental Association, Sekisui Chemicals, Junpukai Health Care Center, Osaka Chamber of Commerce and Industry, non‐financial support from Japan Productivity Center, outside the submitted work.

## AUTHOR CONTRIBUTIONS

YK, KW, DH, KN, YH, MI, KI, and NK contributed substantially to the paper's conception, design, screening and evaluating studies, and writing and approved the manuscript for submission.

## Data Availability

Data sharing not applicable—no new data generated.
